# In Vitro Micropropagation of a Wild Dragon Fruit Accession (*Selenicereus* sp.) with Orange Peel and Fuchsia Pulp from the Amazonas Region, Peru

**DOI:** 10.3390/plants15152327

**Published:** 2026-07-29

**Authors:** José Maximino Saavedra-García, Angel David Hernández-Amasifuen, Alexandra Jherina Pineda-Lázaro, Elvin Delgado-Mera, Jorge Luis Peláez-Rivera, Gilberto Ubaldo Ascón-Dionicio, Orlando Ríos-Ramírez, Elías Torres-Flores, María Emilia Ruíz-Sánchez, Mike Anderson Corazon-Guivin

**Affiliations:** Laboratorio de Biología y Genética Molecular, Universidad Nacional de San Martín, Jr. Amorarca N° 315, Morales 22201, Peru; jmsaavedrag@alumno.unsm.edu.pe (J.M.S.-G.); ahernandeza@unsm.edu.pe (A.D.H.-A.); sherina.97.19@gmail.com (A.J.P.-L.); edelgadom@alumno.unsm.edu.pe (E.D.-M.); jlpelaez@unsm.edu.pe (J.L.P.-R.); guascon@unsm.edu.pe (G.U.A.-D.); orios@unsm.edu.pe (O.R.-R.); etorres@unsm.edu.pe (E.T.-F.); meruiz@unsm.edu.pe (M.E.R.-S.)

**Keywords:** in vitro multiplication, 6-benzylaminopurine, in vitro rooting, indole-3-butyric acid, ex vitro acclimatization, moss

## Abstract

The wild dragon fruit (*Selenicereus* spp.) with orange peel and fuchsia pulp, known as “Naranja de Churuja”, represents a promising plant genetic resource from the Amazonas region of Peru. The objective of this study was to develop an in vitro micropropagation protocol for this local accession by evaluating shoot induction and proliferation, in vitro rooting, and ex vitro acclimatization. During the multiplication phase, nine combinations of 6-benzylaminopurine (BAP) and indole-3-butyric acid (IBA) were evaluated using cladode segments. Shoot induction was high in most treatments (95.56–100.00%), and the combination of 2.0 mg L^−1^ BAP + 0.1 mg L^−1^ IBA produced the highest number of shoots per explant (9.67), whereas 1.0 mg L^−1^ BAP without IBA promoted the greatest shoot elongation (2.34 cm). During the rooting phase, 1.50 mg L^−1^ IBA produced the highest number of roots per explant (3.41), whereas 1.00 mg L^−1^ IBA generated the greatest shoot length (4.12 cm). During ex vitro acclimatization, all plants survived (100% survival), but growth depended significantly on the substrate, with the moss treatment promoting the greatest development. In conclusion, an efficient micropropagation protocol was established for the wild “Naranja de Churuja” accession, with potential for mass propagation, germplasm conservation, and future biotechnological applications, including genetic transformation and genome editing.

## 1. Introduction

Dragon fruit or pitahaya (*Selenicereus* spp.) is an emerging crop of increasing commercial and nutritional importance due to the high acceptance of its fruits in fresh and processed markets [1]. Its fruits are notable for their water content, soluble sugars, vitamin C, and minerals, as well as seeds rich in unsaturated lipids, which increases their value as a healthy food and agro-industrial raw material [2]. They also contain betalains, polyphenols, and flavonoids that contribute to the color, organoleptic quality, and antioxidant capacity of the pulp and peel [3]. Therefore, pitaya represents a promising fruit crop resource with potential for functional foods and high-value-added by-products [4,5].

Despite its growing productive potential, dragon fruit cultivation faces phytosanitary constraints that can compromise plant survival, fruit quality, and the profitability of the production system [6,7]. The main diseases include anthracnose, stem cankers, cladode and fruit rots, and necrotic spots, which reduce the photosynthetic area and affect plant vigor [8,9]. In addition, pests such as mealybugs, aphids, weevils, fruit flies, and nematodes reinforce the need for healthy and vigorous plant material [10,11].

In Peru, production and technical interest are concentrated mainly in Amazonas and San Martín, regions with favorable conditions and a growing appreciation of this crop as a productive alternative for small and medium-sized farmers [12,13]. In addition, these regions contain wild materials with yellow peel and white pulp (*S. megalanthus*), red peel and red pulp (*S. monocanthus*), as well as orange peel and fuchsia pulp (*Selenicereus* sp.), highlighting their phenotypic diversity and regional genetic uniqueness; this suggests the existence of a local genetic reservoir of great interest for selection, conservation, and breeding programs [14,15,16].

The conservation of genetic diversity in dragon fruit is a priority because of the morphological variability among cultivated and wild materials, as well as the processes of local differentiation and hybridization that broaden its genetic base [17]. Molecular studies have shown that these resources possess valuable diversity for breeding and conservation [18]. However, this variability may be eroded by the disappearance of traditional management systems, agricultural simplification, and land-use change [19,20]. Therefore, in situ and ex situ conservation of dragon fruit germplasm is essential to preserve traits associated with adaptation, fruit quality, and tolerance to biotic and abiotic stresses [21].

In response to these limitations, plant biotechnology and, particularly, tissue culture constitute an efficient alternative for clonal propagation, germplasm conservation, and the production of plant material with improved sanitary and uniformity standards [22]. Unlike sexual propagation, which can generate high segregation, and conventional dragon fruit propagation by cuttings, micropropagation enables elite genotypes to be multiplied year-round under controlled conditions [23]. Advances in the in vitro regeneration and multiplication of yellow dragon fruit have been reported, confirming the technical feasibility of developing mass propagation schemes [24,25,26]. Furthermore, the development of effective and transferable protocols contributes not only to production, but also to conservation, sustainable use, and future advanced genetic improvement strategies, such as genetic transformation and genome editing [27,28,29].

Therefore, the objective of the present study was to develop the first in vitro micropropagation protocol for the wild dragon fruit accession with orange peel and fuchsia pulp, “Naranja de Churuja”, from the Amazonas region of Peru, with emphasis on efficient plant regeneration. In this way, the study aimed to generate a methodology that enables the production of healthy, uniform plant material in sufficient quantities, thereby contributing both to the mass propagation of this promising resource and to its conservation and future use in germplasm production and management programs.

## 2. Results

### 2.1. Shoot Induction and Proliferation

**Figure 1:**
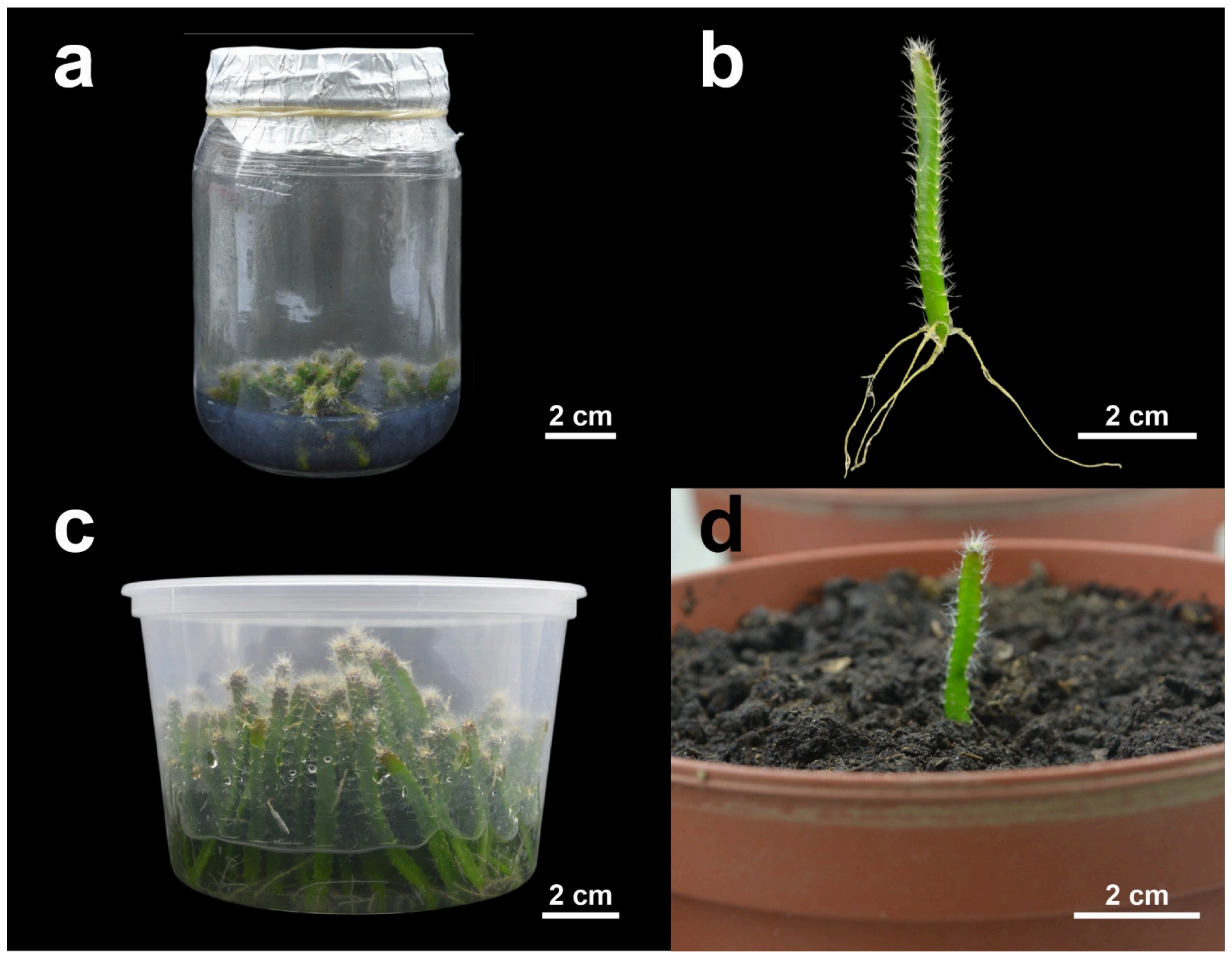
Stages of the in vitro micropropagation and ex vitro transition of “Naranja de Churuja” dragon fruit. (**a**) Shoot proliferation obtained under the best multiplication treatment, consisting of MS medium supplemented with 2.0 mg L^−1^ BAP + 0.1 mg L^−1^ IBA. (**b**) Rooted plantlet obtained during the in vitro rooting phase, corresponding to one of the most favorable IBA treatments (1.5 mg L^−1^). (**c**) Pre-acclimatization of rooted plantlets in plastic boxes containing sterile water for seven days under controlled environmental conditions. (**d**) Initial establishment of plantlets in substrate during the acclimatization phase, before transfer to nursery conditions. Scale bars = 2 cm.

The combination of BAP and IBA significantly affected in vitro regeneration, the number of shoots per explant, and shoot length in “Naranja de Churuja” dragon fruit (*p* < 0.001) (Table 1). Overall, regeneration was high in most treatments, with values ranging from 95.56 to 100.00%, similar to the control, except for 1.0 mg L^−1^ BAP + 0.1 mg L^−1^ IBA, which showed the lowest regeneration percentage, at 75.56%. Regarding the number of shoots per explant, the best treatment was 2.0 mg L^−1^ BAP + 0.1 mg L^−1^ IBA, with 9.67 shoots, clearly exceeding the control, which recorded 2.33 shoots. The treatments with 1.0 mg L^−1^ BAP without IBA, with 5.25 shoots, and, to a lesser extent, 1.5 and 0.5 mg L^−1^ BAP without IBA, with 4.33 and 4.17 shoots, respectively, also stood out. In contrast, treatments with 0.1 mg L^−1^ IBA combined with low and intermediate BAP concentrations showed the lowest values, with means between 1.92 and 2.19 shoots per explant. For shoot length, the highest value was obtained with 1.0 mg L^−1^ BAP without IBA, at 2.34 cm, followed by 0.5 mg L^−1^ BAP without IBA, at 2.09 cm; both were higher than the control, which reached 1.60 cm. Conversely, the lowest elongation was again observed with 1.0 mg L^−1^ BAP + 0.1 mg L^−1^ IBA, at 0.59. These results indicate that the combination of 2.0 mg L^−1^ BAP + 0.1 mg L^−1^ IBA was the most efficient for maximizing shoot proliferation (Figure 1a), whereas 1.0 mg L^−1^ BAP without IBA favored the greatest shoot elongation.

### 2.2. Root Induction

IBA concentration significantly affected in vitro rooting, the number of roots per explant, root length, and shoot length in “Naranja de Churuja” dragon fruit (*p* < 0.001) (Table 2). Overall, rooting percentage was high in most treatments, with values ranging from 97.22 to 100.00%, similar to the control, although the concentrations of 0.20 and 1.00 mg L^−1^ reduced this response to 91.67%. Regarding the number of roots per explant, the best treatment was 1.50 mg L^−1^ IBA (Figure 1b), with 3.41 roots, followed by 1.00 mg L^−1^ with 3.26 and 0.05 mg L^−1^ with 3.15, all higher than the control, which presented 1.78 roots. However, the greatest root length was obtained in the treatment without IBA, at 4.71, without significant differences from 0.05 and 0.10 mg L^−1^, whereas the concentrations of 0.75, 1.00, and 1.50 mg L^−1^ produced shorter roots. For shoot length, the best result was recorded with 1.00 mg L^−1^ IBA, at 4.12 cm, followed by 1.50 mg L^−1^ with 3.72 cm, both higher than the control, which reached 2.70 cm; conversely, 2.00 mg L^−1^ showed the lowest elongation, at 2.06 cm. These results indicate that intermediate IBA concentrations, especially 1.00 and 1.50 mg L^−1^, favored root formation and shoot elongation, whereas the auxin-free treatment promoted longer roots.

### 2.3. Ex Vitro Acclimatization of In Vitro-Micropropagated Plantlets

**Figure 2:**
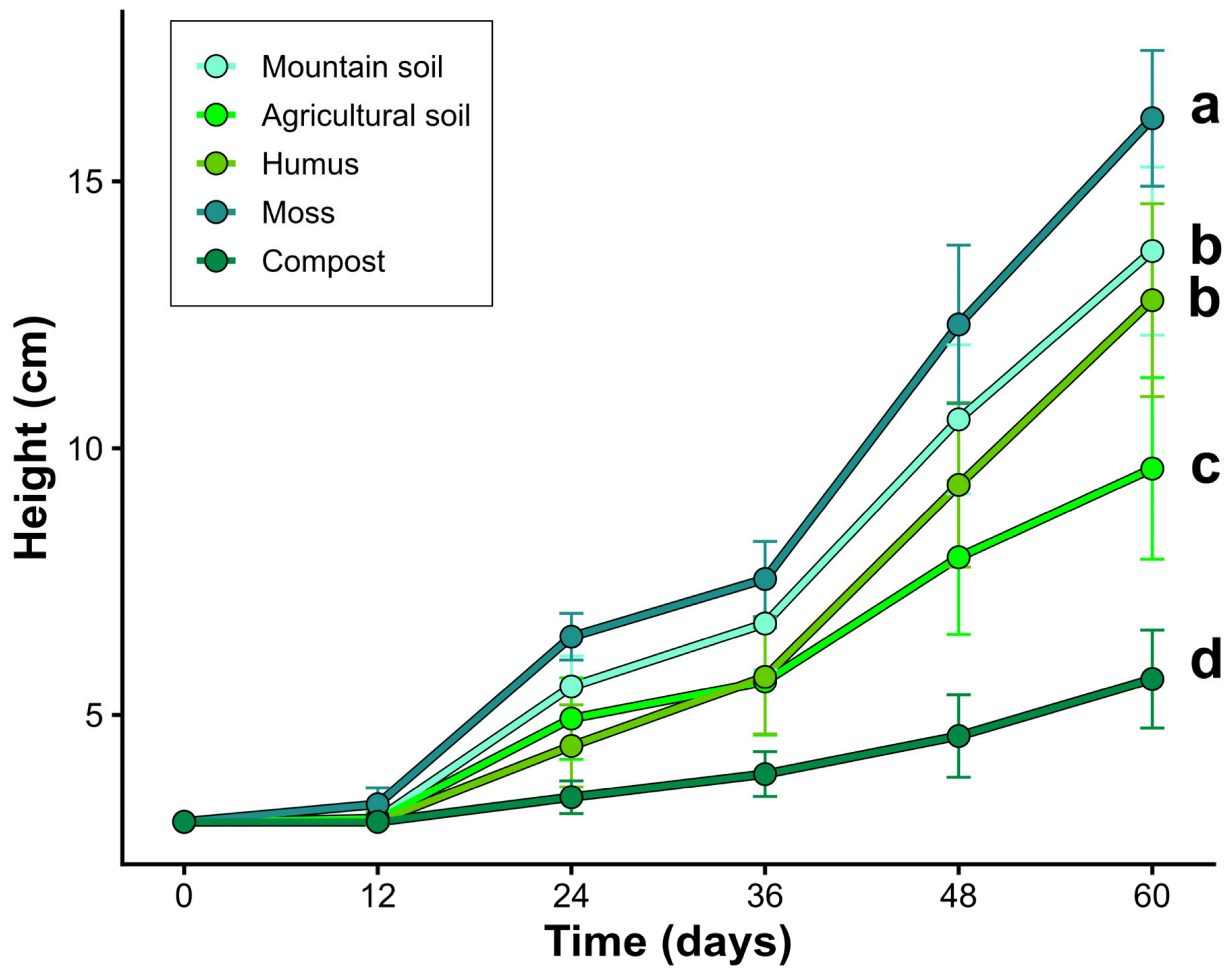
Plant height dynamics during ex vitro acclimatization of “Naranja de Churuja” dragon fruit in different substrates over 60 days. Values represent mean ± standard deviation. Different letters at 60 days indicate significant differences among substrates according to Tukey’s HSD test at *p* ≤ 0.05.

**Figure 3:**
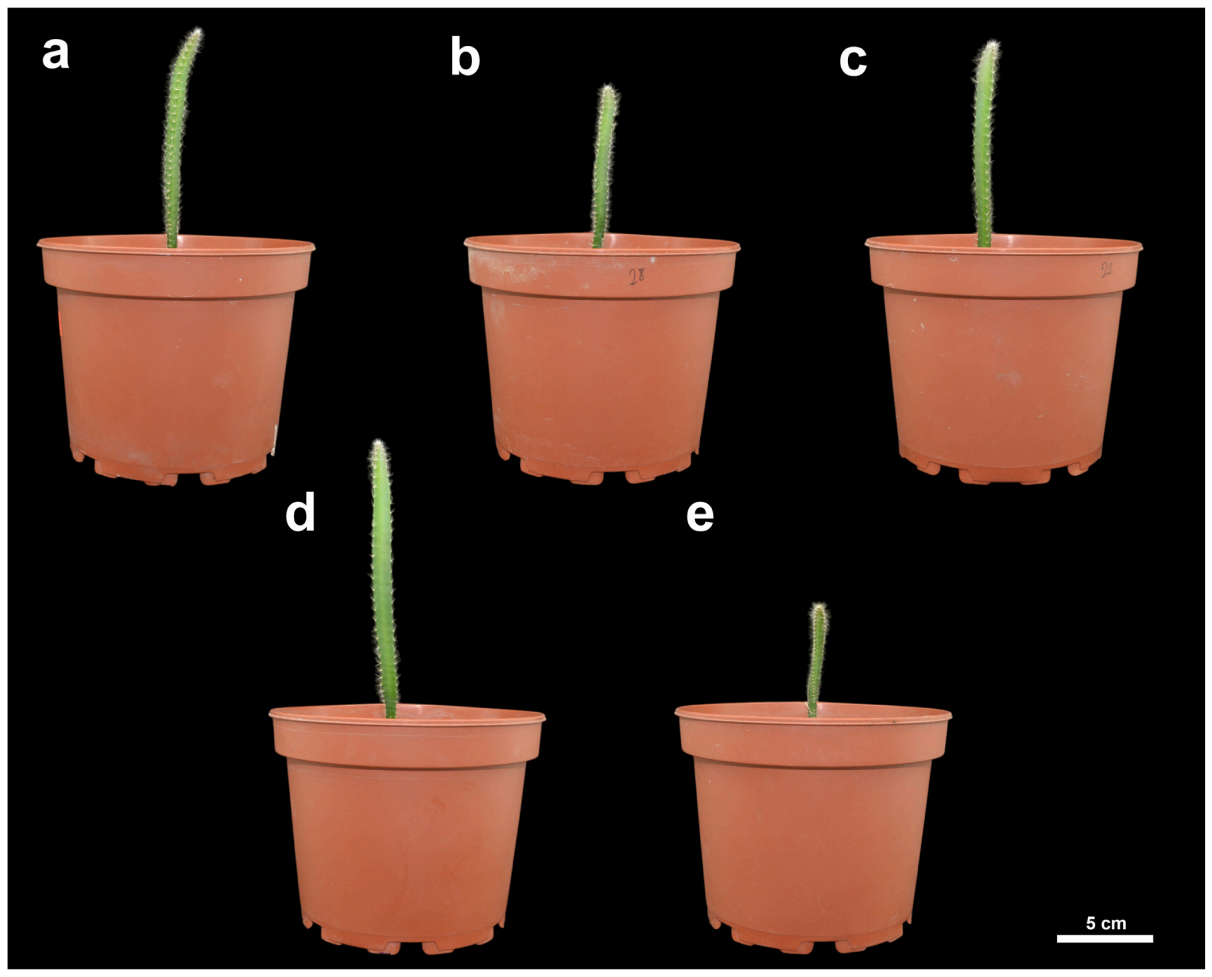
Visual response of “Naranja de Churuja” dragon fruit plantlets after 60 days of ex vitro acclimatization in different substrates. (**a**) Mountain soil, (**b**) agricultural soil, (**c**) humus, (**d**) moss, and (**e**) compost. The images show the differential effect of substrate type on plantlet growth and establishment at the end of the acclimatization period. Scale bar = 5 cm.

During ex vitro acclimatization, substrate type significantly affected plant height over time (*p* < 0.001). Notably, all plants survived throughout the 60-day evaluation period, with a survival rate of 100% in all treatments. Although plants started with similar heights, differences among treatments began to become evident after 24–36 days and became more pronounced at the end of the experimental period (Figure 2). At 60 days, the moss treatment showed the greatest plant height, at 16.18 cm, significantly exceeding all other treatments, followed by mountain soil and humus, with 13.69 and 12.77 cm, respectively, with no statistical differences between them. In contrast, agricultural soil showed an intermediate response, at 9.62 cm, whereas compost recorded the lowest final height, at 5.67 cm. These results indicate that the moss substrate favored the highest growth rate during acclimatization, whereas compost markedly limited the vegetative development of the plants (Figure 3).

Morphological evaluations performed at the end of acclimatization confirmed this trend, with highly significant differences among treatments for cladode diameter, area, volume, number of roots per plant, and root length (*p* < 0.001) (Table 3). The moss treatment showed the highest values for cladode diameter (9.06 cm), area (4.37 cm^2^), and volume (13.85 cm^3^), significantly exceeding the other substrates. It also showed the greatest root length, at 7.91 cm, and one of the highest numbers of roots per plant (5.20), without differing statistically from mountain soil, which also recorded a high number of roots (5.07). The mountain soil and humus treatments showed intermediate values for most variables, whereas agricultural soil showed an intermediate-to-low response, particularly in area and volume. In contrast, compost was the least favorable treatment, recording the lowest values for diameter (4.81 cm), area (0.75 cm^2^), volume (4.53 cm^3^), number of roots (3.80), and root length (4.23). These results confirm that the moss substrate was the most suitable for promoting the ex vitro establishment and growth of “Naranja de Churuja” dragon fruit.

The physicochemical characterization of the substrates used during ex vitro acclimatization showed significant differences among treatments for most of the variables evaluated (*p* < 0.001), whereas pH and N content did not show statistical differences (*p* > 0.05) (Table 4). In terms of texture, humus and compost recorded the highest sand percentages, at 78.03 and 78.81%, respectively, whereas moss was distinguished from the other substrates by its peat texture. Electrical conductivity (EC) showed a contrasting pattern, with the highest values in compost and humus (5.32 and 4.56 dS/m, respectively), significantly higher than those of mountain soil, agricultural soil, and moss, indicating a greater salt load in these substrates. Regarding chemical fertility, humus showed the highest contents of P (12.98 ppm), K (206.07 ppm), cation exchange capacity (CEC) (21.33 meq/100 g), exchangeable Ca^2+^ (17.27 meq/100 g), and exchangeable Mg^2+^ (1.39 meq/100 g), standing out as the substrate with the greatest nutrient availability. In contrast, moss and compost recorded the lowest P and K values, and compost also showed the highest concentration of exchangeable Na^+^ (2.33 meq/100 g), together with high EC, suggesting a potentially restrictive chemical condition. These results show that the substrates differed markedly in their physical and chemical properties, especially in salinity, nutrient availability, and sodium content, factors that probably influenced plantlet response during acclimatization.

**Figure 4:**
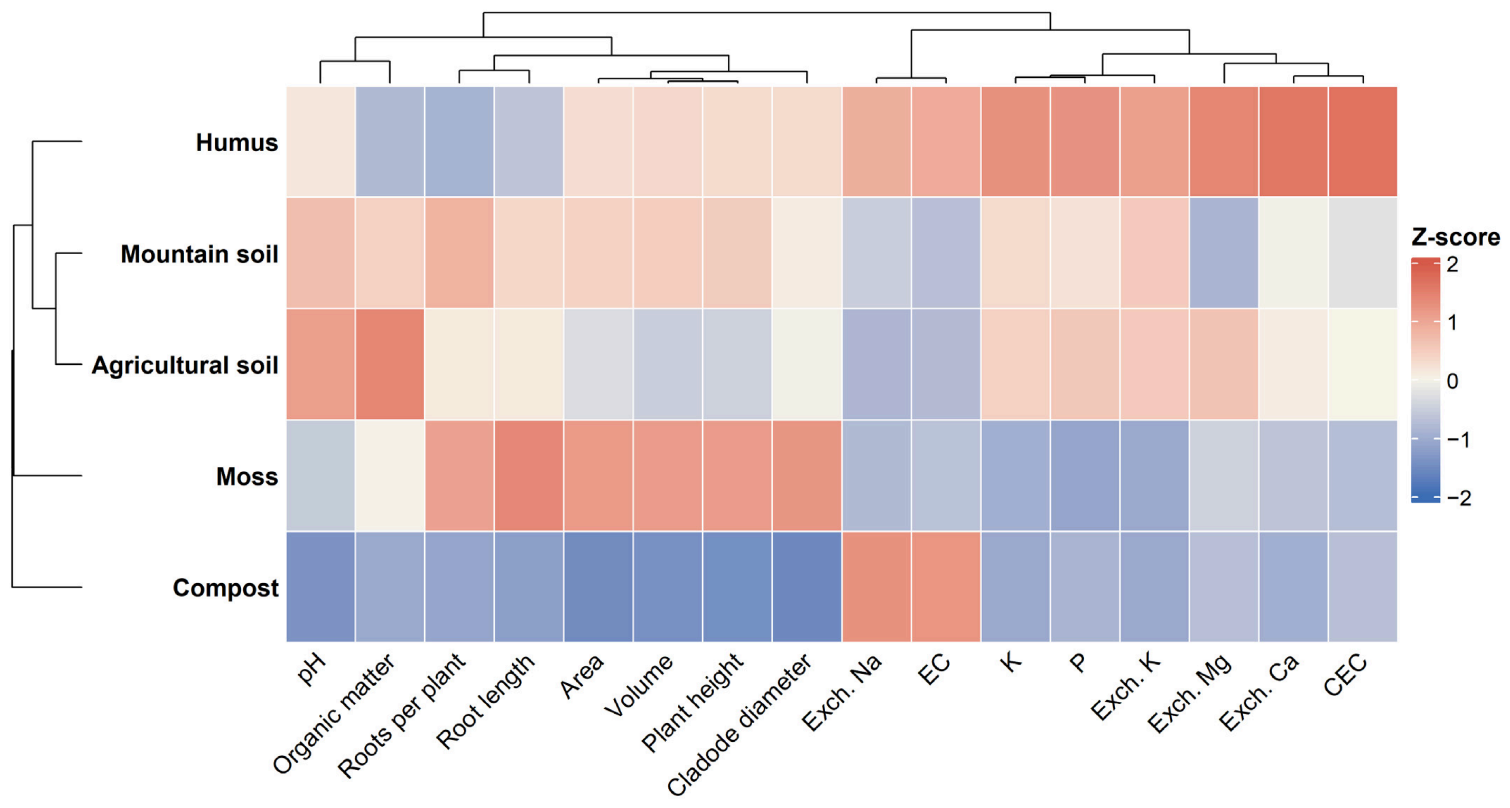
Integrated heatmap of morphological traits and substrate physicochemical properties during the ex vitro acclimatization of “Naranja de Churuja” dragon fruit. Rows represent substrate treatments, and columns represent standardized morphological and physicochemical variables. Color intensity indicates z-score values, with red tones representing higher relative values and blue tones representing lower relative values.

Figure 4 showed a clear separation among substrates according to the integration of morphological and chemical variables. Moss was associated with the highest relative values for plant height, cladode diameter, area, volume, number of roots, and root length, confirming its superior performance during acclimatization. This pattern coincided with lower relative values of EC and exchangeable Na^+^, suggesting a less restrictive environment for growth. In contrast, compost showed the lowest values for most morphological variables and the highest values for EC and exchangeable Na^+^, indicating an unfavorable condition associated with salinity and ionic imbalance. Humus showed high relative concentrations of P, K, Ca, Mg, and CEC, but only intermediate growth performance, whereas mountain soil and agricultural soil showed intermediate responses. The heatmap shows that growth during acclimatization was more closely related to lower salinity and lower Na^+^ than to greater nutrient availability.

## 3. Discussion

The in vitro shoot induction of “Naranja de Churuja” dragon fruit showed high organogenic capacity in most treatments, indicating that the cladode segments used as explants have marked morphogenic competence under suitable culture conditions. The fact that shoot induction remained between 95.56 and 100.00% in almost all combinations suggests that the plant material responded favorably to the basal medium and that BAP addition mainly modulated the intensity of proliferation rather than the intrinsic capacity to induction of shoots. This behavior agrees with previous reports in dragon fruit and other cacti, where the organogenic response is usually high when juvenile explants and moderate cytokinin concentrations are used, although the magnitude of this response depends on genotype, hormonal balance, and explant type [30,31,32,33].

Although shoot induction was generally high, shoot proliferation and elongation showed a much stronger dependence on the balance between BAP and IBA. The combination of 2.0 mg L^−1^ BAP + 0.1 mg L^−1^ IBA produced the highest number of shoots per explant, suggesting that, in this accession, an increase in cytokinin availability accompanied by a low auxin concentration favored meristematic activation and bud multiplication. The marked increase from 1.92 (1.5 mg L^−1^ BAP + 0.1 mg L^−1^) to 9.67 (2.0 mg L^−1^ BAP + 0.1 mg L^−1^) shoots per explant may therefore reflect a threshold response, provided a hormonal balance sufficient to activate multiple pre-existing areolar meristems simultaneously. This behavior is consistent with the physiological role of cytokinins in cell division and in the promotion of adventitious shooting, as well as with the need for a specific hormonal balance to maximize proliferation in succulent and cactus species [34,35,36]. However, the optimal treatment for elongation was 1.0 mg L^−1^ BAP without IBA, indicating that maximum proliferation does not necessarily coincide with the greatest longitudinal shoot growth. This dissociation between number and length has been reported in other dragon fruit studies, where high concentrations of regulators can induce numerous shoots but with reduced elongation or a more compact architecture, making it necessary to prioritize either mass multiplication or individual shoot quality depending on the objective of the protocol [25,26,37].

The lower performance observed with the combination of 1.0 mg L^−1^ BAP + 0.1 mg L^−1^ IBA, which simultaneously reduced shoot induction, number of shoots, and shoot length, shows that the response of this accession did not follow a linear trend in relation to increasing regulator concentrations, but was instead subject to specific hormonal interactions. In in vitro culture systems, small variations in the cytokinin-to-auxin ratio can shift the response from organogenesis toward callogenesis or even induce less vigorous, compact, or physiologically less functional shoots [38,39,40]. From an applied perspective, this finding is important because it indicates that the objective of maximizing shoot production should not compromise the morphogenic quality of the induction material. In addition, in protocols aimed at commercial clonal propagation, it is advisable to minimize indirect responses via callus to reduce the risk of somaclonal variation, especially in genotypes of local agronomic interest for which faithful conservation of morphological and productive attributes is sought [41,42,43]. Therefore, the combination of 2.0 mg L^−1^ BAP + 0.1 mg L^−1^ IBA can be considered the most efficient for multiplication, whereas 1.0 mg L^−1^ BAP without IBA represents a more suitable option when shoot elongation and vigor are prioritized.

During the rooting phase, the high response observed in most treatments confirmed that “Naranja de Churuja” dragon fruit has a high in vitro rhizogenic capacity, a characteristic consistent with reports for other dragon fruit species and cultivars [32,34]. However, the response was again not uniform across all variables, as intermediate IBA concentrations favored the formation of a greater number of roots and greater shoot length, whereas the auxin-free treatment produced longer roots. This differentiation suggests that exogenous auxin primarily stimulated the initiation and multiplication of root primordia, but not necessarily their subsequent elongation, a behavior that has been documented in other dragon fruit propagation systems, where IBA promotes abundant rooting and vigorous explants, although maximum root length can be achieved in media with a lower hormonal load or even without supplemental auxin [34,37,44].

To obtain plants with better overall architecture, IBA concentrations of 1.00 and 1.50 mg L^−1^ were more suitable, as they combined a good rooting response, a higher number of roots, and greater shoot length. Although the control produced longer roots, its lower number of roots limits its advantage from an acclimatization perspective, since ex vitro establishment depends not only on root length, but also on the number of active absorption points and the overall capacity of the root system to resume water and nutrient uptake under less controlled conditions [45,46,47]. This interpretation agrees with the fact that, in micropropagation, IBA-induced roots are usually functional and compatible with subsequent acclimatization, even when they do not reach the greatest absolute length, provided that the shoot remains vigorous and does not show malformations [37,44]. Consequently, the rooting phase of the present study not only confirmed the usefulness of IBA as the main auxin for this accession, but also allowed the identification of a hormonal range capable of generating plants better prepared for the ex vitro transition. From a practical and economic perspective, the auxin-free medium may be preferable for routine propagation because it achieved 100% rooting without the additional cost of IBA and produced longer roots and shoots. Conversely, 2.00 mg L^−1^ IBA increased root number but reduced longitudinal growth, and its use would therefore be justified only when a greater number of root initiation sites is considered more important than root and shoot elongation.

The acclimatization phase confirmed that the in vitro-regenerated and rooted material maintained high physiological quality, since all plants survived throughout the 60-day evaluation period. This 100% survival agrees with several reports in dragon fruit and demonstrates that, under adequate management, transfer from in vitro conditions to the nursery can be performed without major losses, even in genetically diverse materials or materials previously exposed to different hormonal combinations [26,30,34,44,48]. However, the absence of mortality did not imply equivalence in plant quality, as aerial and root growth clearly differentiated the substrates. The moss treatment produced the greatest height, cladode diameter, area, volume, number of roots, and root length, outperforming the other substrates and showing that, in this accession, the best ex vitro establishment was associated with a physical environment favorable for root expansion and, at the same time, less restrictive in chemical terms. These results were also comparatively superior to several acclimatization reports in *S. costaricensis*, *S. monacanthus*, *S. polyrhizus*, and *S. megalanthus*, highlighting the potential of the moss substrate for this local accession [25,26,31,48].

The physicochemical characterization of the substrates and, above all, the integrated patterns observed in the heatmap clearly showed that plant response during acclimatization was more closely associated with salinity and exchangeable Na^+^ than with greater nutrient availability in the substrate. The most illustrative case was humus, which presented the highest contents of P, K, CEC, Ca^2+^, and Mg^2+^, but induced only intermediate growth; in contrast, moss, despite showing relatively low levels of several nutrients, produced the best morphological performance. This pattern was further reinforced by compost, which showed the highest EC and the highest exchangeable Na^+^, while simultaneously recording the lowest values for all growth variables. From a physiological standpoint, this response is fully consistent with the restrictive effect of salinity and sodium on plant growth [49]. High EC reduces the osmotic potential of the substrate and limits water uptake, whereas excess Na^+^ alters ionic balance, competes with other essential cations, and can compromise cell expansion, differentiation, and root development processes. Although Na^+^ can assume partial or complementary functions when K is limiting, this potential benefit depends on moderate concentrations and an ionic balance that was not observed in compost, where the combination of high EC and high Na^+^ coincided with the poorest growth [50]. In contrast, the better performance of moss suggests that a substrate with a lower salt load and better physical condition can compensate for lower initial chemical richness and favor a more efficient recovery of water-related and root functions during the ex vitro transition [49,50,51,52]. This result is also consistent with observations in other studies of acclimatization of micropropagated species, where substrate physical quality, aeration, and effective water availability are decisive during the first weeks of hardening [53,54].

## 4. Materials and Methods

### 4.1. Plant Material and Culture Establishment

**Figure 5:**
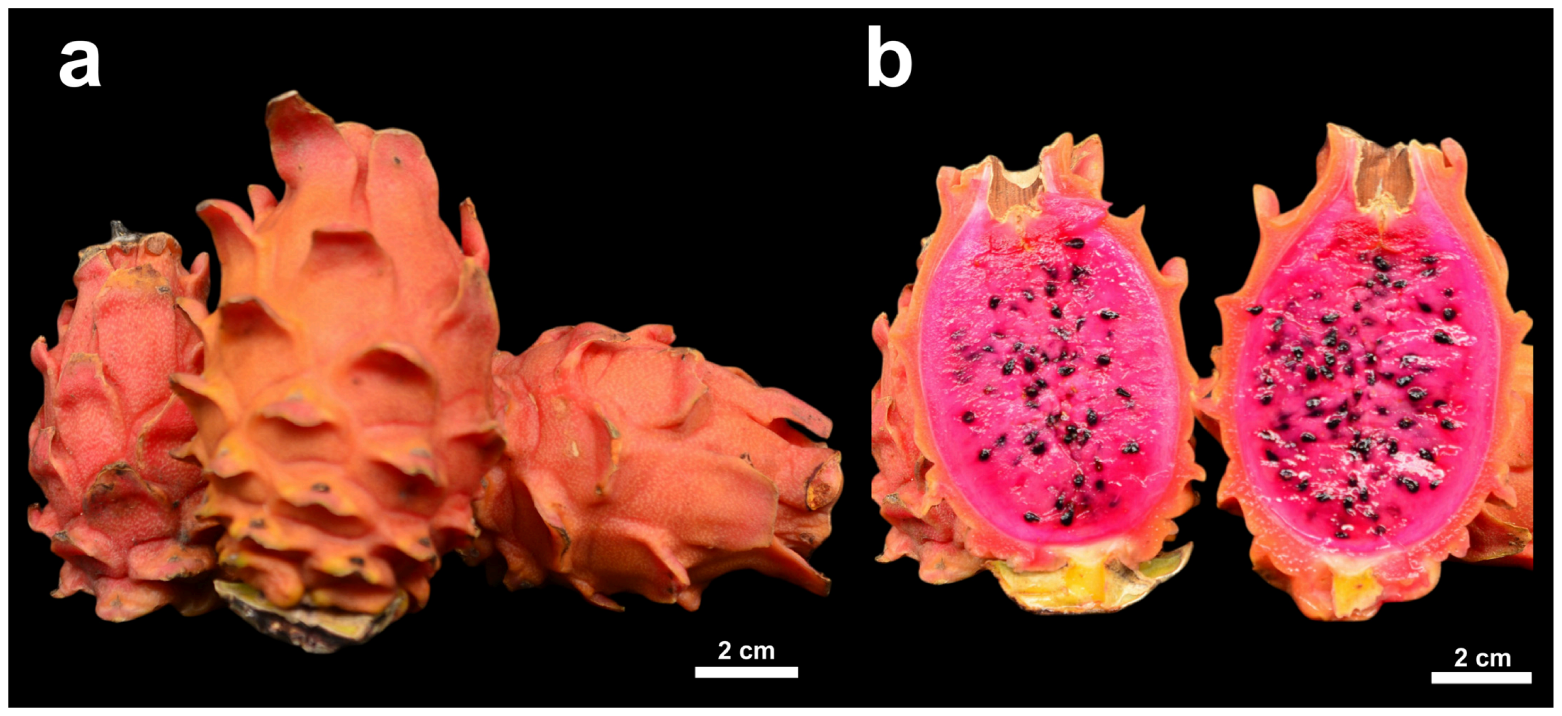
Morphological characteristics of the wild dragon fruit accession “Naranja de Churuja” (*Selenicereus* sp.). (**a**) External appearance of mature fruits showing the characteristic orange peel. (**b**) Longitudinal section of the fruit showing the fuchsia pulp and the distribution of black seeds. Scale bars = 2 cm.

Dragon fruit (*Selenicereus* sp.) with orange peel and fuchsia pulp, locally known as “Naranja de Churuja” (Figure 5), was obtained from self-pollinated flowers and harvested from the dragon fruit germplasm bank of the Laboratorio de Biología y Genética Molecular (LBGM), Universidad Nacional de San Martín, located in the district of Morales, province of San Martín, department of San Martín, Peru (06°35′28″ S, 76°18′47″ W), at an altitude of 230 m a.s.l. Seeds were collected from the pulp and sterilized by first immersing them in 70% (*v*/*v*) ethanol for 60 s and then in 2% (*v*/*v*) sodium hypochlorite for 10 min. The seeds were then rinsed three times with sterile distilled water. Under aseptic conditions, the seeds were cultured on half-strength MS medium [55] supplemented with 2% (*w*/*v*) sucrose and 0.6% (*w*/*v*) agar, with the pH adjusted to 5.8. Cultures were incubated in a growth room at 25 ± 1 °C with a 16/8 h (light/dark) photoperiod under fluorescent light at 80 μmol m^−2^ s^−1^. All experiments were conducted in the plant growth area of the LBGM.

### 4.2. Shoot Induction and Proliferation

After approximately three months of in vitro growth, seedlings that had reached approximately 4 cm in height and showed vigorous development were selected as explant sources. The juvenile cladodes of the selected seedlings were excised into 0.5 cm-long segments and cultured on MS medium supplemented with 6-benzylaminopurine (BAP) at 0.5, 1.0, 1.5, and 2.0 mg L^−1^, either alone or in combination with 0.1 mg L^−1^ indole-3-butyric acid (IBA). In addition, a control treatment consisting of MS medium without growth regulators was included. In total, nine treatments were evaluated. All treatments were prepared with 3% (*w*/*v*) sucrose, 0.2% (*w*/*v*) activated charcoal, and 0.6% (*w*/*v*) agar, with the pH adjusted to 5.8. Cultures were incubated at 25 ± 1 °C under a light intensity of 80 μmol m^−2^ s^−1^ with a 16/8 h light/dark photoperiod. After eight weeks of culture, the regeneration percentage, number of shoots per explant, and shoot length were evaluated.

### 4.3. Root Induction

Shoots obtained during the multiplication phase were individualized (1.5 cm in length) and cultured on MS medium supplemented with IBA at concentrations of 0.05, 0.10, 0.20, 0.50, 0.75, 1.00, 1.50, and 2.00 mg L^−1^. In addition, a control treatment consisting of MS medium without growth regulators was included. In total, nine treatments were evaluated. All treatments were prepared with 3% (*w*/*v*) sucrose, 0.2% (*w*/*v*) activated charcoal, and 0.6% (*w*/*v*) agar, with the pH adjusted to 5.8. Cultures were incubated at 25 ± 1 °C under a light intensity of 80 μmol m^−2^ s^−1^ with a 16/8 h light/dark photoperiod. After eight weeks of culture, rooting percentage, number of roots per explant, root length, and shoot length were evaluated.

### 4.4. Ex Vitro Acclimatization of In Vitro-Micropropagated Plantlets

Rooted plants obtained in vitro were carefully removed from the culture vessels, and their roots were gently washed with sterile distilled water to completely remove agar residues. The plants were then placed in plastic boxes containing sterile water (the entire root system submerged) and maintained for seven days under controlled conditions of 25 ± 1 °C, a light intensity of 80 μmol m^−2^ s^−1^, and a 16/8 h light/dark photoperiod as a pre-acclimatization phase [34].

After this stage, plantlets with an approximate shoot length of 3 cm and at least three developed roots were selected to standardize the initial plant material. The plantlets were transferred to pots containing organic substrates. Five treatments were evaluated: mountain soil, agricultural soil, humus, moss, and compost. The plants remained for an additional 12 days under the same controlled environmental conditions and were then transferred to the nursery under natural temperature and photoperiod. Irrigation was performed every two days. Survival rate and cladode length were evaluated at 12-day intervals until 60 days of nursery acclimatization were completed. At the end of this period, cladode diameter, number of roots, root length, volume, and area were also evaluated. Cladode volume was determined by water displacement according to Archimedes’ principle, whereas cladode area was quantified from digital images using ImageJ (version 1.54p) software, following the procedure described by Lee and Chang [31]. In addition, before planting in pots, the physicochemical characterization of the substrates was carried out.

### 4.5. Experimental Design and Statistical Analysis

All experiments were conducted under a completely randomized design (CRD). In the in vitro multiplication phase, nine treatments were evaluated, with nine replicates per treatment, considering a flask containing four cladode segments as the experimental unit. In the in vitro rooting phase, nine treatments were also evaluated, with nine replicates per treatment, with the experimental unit consisting of a flask containing three individualized shoots. In the ex vitro acclimatization phase, five substrate treatments were evaluated, with 15 replicates per treatment; in this case, the experimental unit corresponded to one pot containing one plant.

Statistical analysis was performed using R (version 4.5.1 for Windows). Before inferential analysis, the assumptions of normality and homogeneity of variances were verified using the Shapiro–Wilk and Levene tests, respectively. Subsequently, quantitative variables were analyzed by one-way analysis of variance (ANOVA), and differences among means were determined using Tukey’s HSD test (α = 0.05). The car and agricolae packages were used for data processing.

To integrate plant morphological responses and substrate chemical properties during ex vitro acclimatization, a heatmap was performed. For the heatmap, quantitative variables were standardized using z-score transformation. The heatmap was generated using the ComplexHeatmap (version 2.26.1) and circlize (version 0.4.17) packages.

## 5. Conclusions

The present study established an efficient micropropagation protocol for the wild dragon fruit “Naranja de Churuja”, demonstrating that the response of the plant material clearly depended on each phase of the process. In the multiplication stage, the combination of 2.0 mg L^−1^ BAP + 0.1 mg L^−1^ IBA was the most efficient for maximizing shoot proliferation, whereas 1.0 mg L^−1^ BAP without IBA favored the greatest elongation. During the rooting phase, intermediate IBA concentrations, particularly 1.0 and 1.5 mg L^−1^, promoted a more suitable root architecture by increasing the number of roots and shoot growth, although the auxin-free treatment produced longer roots. During ex vitro acclimatization, the moss substrate was clearly superior, recording 100% survival and the highest values for height, diameter, area, volume, and root development, confirming it as the best option for establishing micropropagated plants of this local accession. In addition, this system constitutes a platform with potential for future biotechnological applications, including germplasm conservation, mass propagation of local accession, genetic transformation, genome editing, and functional studies, thereby expanding the possibilities for the use and improvement of this plant genetic resource.

## Figures and Tables

**Table 1:** Effect of different BAP and IBA concentrations on the in vitro multiplication of “Naranja de Churuja” dragon fruit after 8 weeks of culture.

BAP (mg L^−1^)	IBA (mg L^−1^)	Shoot Induction (%)	No. of Shoots Per Explant	Shoot Length (cm)
0.0	0.0	100.00 ^a^	2.33 ± 0.42 ^d^	1.60 ± 0.26 ^c^
0.5	0.0	100.00 ^a^	4.17 ± 0.53 ^bc^	2.09 ± 0.20 ^b^
1.0	0.0	100.00 ^a^	5.25 ± 0.61 ^b^	2.34 ± 0.28 ^a^
1.5	0.0	97.78 ^a^	4.33 ± 0.42 ^bc^	1.30 ± 0.19 ^d^
2.0	0.0	95.56 ^a^	4.08 ± 0.53 ^c^	0.97 ± 0.17 ^e^
0.5	0.1	95.56 ^a^	2.19 ± 0.27 ^d^	0.78 ± 0.12 ^f^
1.0	0.1	75.56 ^b^	1.97 ± 0.38 ^d^	0.59 ± 0.16 ^g^
1.5	0.1	97.78 ^a^	1.92 ± 0.33 ^d^	0.80 ± 0.15 ^f^
2.0	0.1	100.00 ^a^	9.67 ± 1.81 ^a^	1.50 ± 0.20 ^c^

Means ± SD followed by different letters are significantly different according to Tukey’s test (*p* ≤ 0.05).

**Table 2:** Effect of different IBA concentrations on root induction in shoots of “Naranja de Churuja” dragon fruit after 8 weeks of culture.

IBA (mg L^−1^)	Rooting (%)	No. of Roots Per Explant	Root Length (cm)	Shoot Length (cm)
0.00	100.00 ^a^	1.78 ± 0.37 ^d^	4.71 ± 0.36 ^a^	2.70 ± 0.29 ^d^
0.05	100.00 ^a^	3.15 ± 0.38 ^ab^	4.33 ± 0.48 ^a^	3.53 ± 0.23 ^bc^
0.10	97.22 ^a^	2.52 ± 0.41 ^c^	4.40 ± 0.62 ^a^	3.59 ± 0.30 ^bc^
0.20	91.67 ^b^	2.85 ± 0.38 ^bc^	4.10 ± 0.91 ^ab^	3.24 ± 0.21 ^c^
0.50	100.00 ^a^	2.74 ± 0.36 ^bc^	3.40 ± 0.53 ^bc^	3.39 ± 0.21 ^bc^
0.75	100.00 ^a^	2.85 ± 0.53 ^bc^	2.91 ± 0.48 ^c^	3.52 ± 0.27 ^bc^
1.00	91.67 ^b^	3.26 ± 0.33 ^ab^	2.93 ± 0.42 ^c^	4.12 ± 0.32 ^a^
1.50	97.22 ^a^	3.41 ± 0.36 ^a^	3.21 ± 0.68 ^c^	3.72 ± 0.31 ^b^
2.00	100.00 ^a^	3.04 ± 0.39 ^abc^	3.26 ± 0.65 ^bc^	2.06 ± 0.19 ^e^

Means ± SD followed by different letters are significantly different according to Tukey’s test (*p* ≤ 0.05).

**Table 3:** Effect of different substrates on the ex vitro acclimatization of “Naranja de Churuja” dragon fruit plantlets after 60 days.

Treatment	Diameter (cm)	Area (cm^2^)	Volume (cm^3^)	No. of Roots Per Plant	Root Length (cm)
Mountain soil	7.31 ± 0.75 ^b^	3.36 ± 0.81 ^b^	11.46 ± 2.43 ^b^	5.07 ± 0.80 ^a^	6.44 ± 1.37 ^b^
Agricultural soil	7.09 ± 0.50 ^b^	2.36 ± 0.78 ^c^	7.91 ± 1.69 ^c^	4.60 ± 0.99 ^ab^	6.11 ± 1.21 ^bc^
Humus	7.65 ± 0.85 ^b^	3.16 ± 0.74 ^b^	11.03 ± 2.26 ^b^	3.93 ± 0.96 ^b^	5.07 ± 0.79 ^cd^
Moss	9.06 ± 0.83 ^a^	4.37 ± 0.75 ^a^	13.85 ± 2.48 ^a^	5.20 ± 0.94 ^a^	7.91 ± 1.37 ^a^
Compost	4.81 ± 0.75 ^c^	0.75 ± 0.22 ^d^	4.53 ± 0.97 ^d^	3.80 ± 0.68 ^b^	4.23 ± 0.74 ^d^

Means ± SD followed by different letters are significantly different according to Tukey’s test (*p* ≤ 0.05).

**Table 4:** Physicochemical characteristics of the substrates used during the ex vitro acclimatization of “Naranja de Churuja” dragon fruit.

Characteristics	Treatment				
	Mountain Soil	Agricultural Soil	Humus	Moss	Compost
Sand (%)	57.05 ± 3.45 ^b^	58.83 ± 4.18 ^b^	78.03 ± 2.49 ^a^	0.00 ± 0.00 ^c^	78.81 ± 1.37 ^a^
Clay (%)	15.81 ± 2.11 ^b^	21.82 ± 3.06 ^a^	12.50 ± 0.67 ^bc^	0.00 ± 0.00 ^d^	10.24 ± 1.01 ^c^
Silt (%)	27.14 ± 4.68 ^a^	19.36 ± 4.04 ^a^	9.47 ± 1.82 ^b^	0.00 ± 0.00 ^c^	10.96 ± 2.38 ^b^
Textural class	Sandy	Sandy	Sandy	Peat	Sandy
pH	6.98 ± 0.22 ^a^	7.07 ± 0.37 ^a^	6.86 ± 0.11 ^a^	6.71 ± 0.34 ^a^	6.53 ± 0.35 ^a^
EC (dS/m)	0.47 ± 0.05 ^b^	0.30 ± 0.05 ^c^	4.56 ± 0.44 ^a^	0.54 ± 0.05 ^b^	5.32 ± 0.60 ^a^
Organic matter (%)	2.79 ± 0.39 ^a^	3.27 ± 0.17 ^a^	2.19 ± 0.15 ^b^	2.57 ± 0.55 ^ab^	2.05 ± 0.08 ^b^
N (%)	0.12 ± 0.02 ^a^	0.11 ± 0.01 ^a^	0.12 ± 0.02 ^a^	0.11 ± 0.01 ^a^	0.12 ± 0.01 ^a^
P (ppm)	10.76 ± 0.53 ^b^	11.52 ± 0.50 ^ab^	12.98 ± 0.64 ^a^	7.90 ± 0.61 ^c^	8.41 ± 0.58 ^c^
K (ppm)	159.97 ± 18.37 ^b^	166.23 ± 19.00 ^b^	206.07 ± 5.91 ^a^	99.01 ± 17.39 ^c^	94.30 ± 7.18 ^c^
CEC (meq/100 g)	13.33 ± 1.53 ^b^	14.33 ± 3.06 ^b^	21.33 ± 2.52 ^a^	11.20 ± 3.27 ^b^	11.33 ± 1.53 ^b^
Exchangeable Ca^2+^ (meq/100 g)	11.31 ± 0.94 ^b^	11.66 ± 2.79 ^b^	17.27 ± 2.07 ^a^	9.06 ± 2.50 ^b^	7.92 ± 1.43 ^b^
Exchangeable Mg^2+^ (meq/100 g)	0.76 ± 0.16 ^c^	1.17 ± 0.13 ^ab^	1.39 ± 0.12 ^a^	0.87 ± 0.10 ^bc^	0.80 ± 0.17 ^c^
Exchangeable K^+^ (meq/100 g)	0.43 ± 0.06 ^a^	0.43 ± 0.06 ^a^	0.50 ± 0.06 ^a^	0.23 ± 0.06 ^b^	0.23 ± 0.06 ^b^
Exchangeable Na^+^ (meq/100 g)	1.07 ± 0.15 ^b^	0.83 ± 0.25 ^b^	2.07 ± 0.15 ^a^	0.87 ± 0.32 ^b^	2.33 ± 0.25 ^a^

Means ± SD followed by different letters are significantly different according to Tukey’s test (*p* ≤ 0.05).

## Data Availability

The datasets used and analyzed in this study are available from the corresponding author upon reasonable request.
